# Comparative Evaluation of [^99m^Tc]Tilmanocept for Sentinel Lymph Node Mapping in Breast Cancer Patients: Results of Two Phase 3 Trials

**DOI:** 10.1245/s10434-013-2887-8

**Published:** 2013-03-17

**Authors:** Anne M. Wallace, Linda K. Han, Stephen P. Povoski, Kenneth Deck, Schlomo Schneebaum, Nathan C. Hall, Carl K. Hoh, Karl K. Limmer, Helen Krontiras, Thomas G. Frazier, Charles Cox, Eli Avisar, Mark Faries, Dennis W. King, Lori Christman, David R. Vera

**Affiliations:** 1 Divisions of Surgical Oncology and Plastic Surgery, UCSD Moores Cancer Center, University of California, San Diego, La Jolla, CA USA; 2 Department of Surgery, Indiana University Simon Breast Center, Indianapolis, IN USA; 3 Department of Surgery, Wexner Medical Center of the Ohio State University, Columbus, OH USA; 4 Department of Surgery, South Orange County Medical, Laguna Hills, CA USA; 5 Department of Surgery, Sourasky Medical Center, Tel Aviv, Israel; 6 Division of Nuclear Medicine, Wexner Medical Center of the Ohio State University, Columbus, OH USA; 7 Division of Nuclear Medicine, UCSD Moores Cancer Center, and the UCSD In Vivo Cancer & Molecular Imaging Program, University of California, San Diego, La Jolla, CA USA; 8 Department of Surgery, The UCSD Moores Cancer Center, University of California, San Diego, La Jolla, CA USA; 9 Department of Surgery, University of Alabama, Birmingham, AL USA; 10 Department of Surgery, Bryn Mawr Hospital, Bryn Mawr, PA USA; 11 Department of Surgery, University of South Florida, Tampa, FL USA; 12 Department of Surgery, University of Miami Hospital, Miami, FL USA; 13 Department of Surgery and Melanoma Research Program, John Wayne Cancer Institute, Santa Monica, CA USA; 14 STATKING Clinical Services, Fairfield, CA USA

## Abstract

**Background:**

Sentinel lymph node (SLN) surgery is used worldwide for staging breast cancer patients and helps limit axillary lymph node dissection. [^99m^Tc]Tilmanocept is a novel receptor-targeted radiopharmaceutical evaluated in 2 open-label, nonrandomized, within-patient, phase 3 trials designed to assess the lymphatic mapping performance.

**Methods:**

A total of 13 centers contributed 148 patients with breast cancer. Each patient received [^99m^Tc]tilmanocept and vital blue dye (VBD). Lymph nodes identified intraoperatively as radioactive and/or blue stained were excised and histologically examined. The primary endpoint, concordance (lower boundary set point at 90 %), was the proportion of nodes detected by VBD and [^99m^Tc]tilmanocept.

**Results:**

A total of 13 centers contributed 148 patients who were injected with both agents. Intraoperatively, 207 of 209 nodes detected by VBD were also detected by [^99m^Tc]tilmanocept for a concordance rate of 99.04 % (*p* < 0.0001). [^99m^Tc]tilmanocept detected a total of 320 nodes, of which 207 (64.7 %) were detected by VBD. [^99m^Tc]Tilmanocept detected at least 1 SLN in more patients (146) than did VBD (131, *p* < 0.0001). In 129 of 131 patients with ≥1 blue node, all blue nodes were radioactive. Of 33 pathology-positive nodes (18.2 % patient pathology rate), [^99m^Tc]tilmanocept detected 31 of 33, whereas VBD detected only 25 of 33 (*p* = 0.0312). No pathology-positive SLNs were detected exclusively by VBD. No serious adverse events were attributed to [^99m^Tc]tilmanocept.

**Conclusion:**

[^99m^Tc]Tilmanocept demonstrated success in detecting a SLN while meeting the primary endpoint. Interestingly, [^99m^Tc]tilmanocept was additionally noted to identify more SLNs in more patients. This localization represented a higher number of metastatic breast cancer lymph nodes than that of VBD.

Sentinel lymph node (SLN) identification during breast surgery has been extensively validated.^1^^–^ 6 In current SLN biopsy practice, a vital blue dye (VBD) may be paired with a colloidal radiotracer with the intent to complement its performance by increasing SLN detection rate and identification accuracy.^7^^,^ 8 However, the overall efficacy of SLN identification depends heavily on the specificity of the agent(s) used in the mapping procedure in order to provide reliable localization and retention of the agent in the sentinel node(s).

[^99m^Tc]Tilmanocept (Fig. 1) is a novel, engineered radiopharmaceutical specifically designed for lymphoscintigraphy and intraoperative SLN detection.^9^ [^99m^Tc]Tilmanocept consists of multiple DTPA and mannose moieties tethered to a dextran scaffold. DTPA chelates and holds ^99m^Tc, while its multiple mannose moieties facilitate specific multivalent binding to mannose receptors (CD206) expressed on the surfaces of reticuloendothelial cells residing within lymph nodes.^10^ Preclinical studies and several phase 1 and phase 2 clinical trials demonstrated that [^99m^Tc]tilmanocept’s chemical structure and relatively small molecular size (MW = 16.7 kDa) and small molecular diameter of 7.1 nm enable [^99m^Tc]tilmanocept to exit its injection site more rapidly than radiolabeled colloids and quickly accumulate at the SLNs, while specific multivalent interactions between its mannose moieties and CD206 enable avid binding to target receptors and retention in SLNs for up to 30 h, without observed transit to second-echelon lymph nodes.^9^^–^ 15

**Fig. 1:**
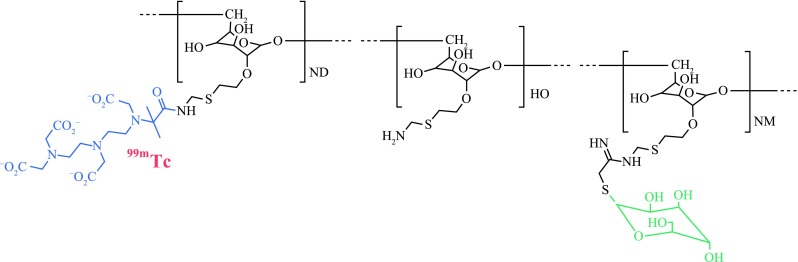
[^99m^Tc]Tilmanocept is composed of a dextran backbone (*black*) to which are attached multiple units of mannose (*gree*n) and DTPA (*blue*). The mannose units provide a molecular mechanism by which [^99m^Tc]tilmanocept avidly binds to a receptor specific to recticuloendothelial cells (CD206), and the DTPA units provide a highly stable means to radiolabel tilmanocept with technetium-99m (*red*). The molecular weight of [^99m^Tc]tilmanocept is approximately 17,000 grams per mole; the molecular diameter is 7.1 nm

The clinical trials reported here were designed in consultation with the US Food and Drug Administration and the European Medicine Agency for the application of [^99m^Tc]tilmanocept for approval as a new drug. Here, we report the results from 2 highly similar prospective, phase 3 clinical trials of [^99m^Tc]tilmanocept in patients undergoing SLN mapping for breast cancer. The results from the melanoma patients are reported separately in a companion paper.^16^ On March 13, 2013 the US Food and Drug Administration approved [^99m^Tc]tilmanocept for intraoperative lymph node mapping.

## Materials and Methods

### Study Design

From June 2008 to April 2011, 2 highly similar phase 3 trials were performed on breast cancer patients without presurgical evidence of lymph node metastases who were scheduled for surgeries that included clinically indicated SLN mapping. The primary aim of both studies was to evaluate the concordance of [^99m^Tc]tilmanocept with VBD, a mapping agent that is routinely used to identify SLNs. The studies were designed as prospective, phase 3, open-label, nonrandomized, within-patient trials in which all patients received both [^99m^Tc]tilmanocept and a VBD (Lymphazurin). The first trial involved 8 centers enrolling at least 1 breast cancer patient between June 2008 and June 2009. A second trial was conducted to extend the safety database to 500 subjects (including phase 1 and 2 trials). The second trial involved 5 sites enrolling breast cancer patients from July 2010 to April 2011 and used the same study design and procedures. These studies were powered at 0.8 or greater for the primary endpoint. All patients gave written informed consent. The Western Institutional Review Board and institutional review boards at each enrolling institution approved the protocol, patient instructions, and informed consent documentation. Both studies complied with all provisions of the Declaration of Helsinki and US laws requiring registration and updates via ClinicalTrials.gov (trial No. NCT00671918 and NCT01106040). This report describes results obtained from the breast cancer patients who participated in these studies.

Enrollment criteria included the histologically confirmed presence of unilateral breast cancer with a surgical treatment plan that included SLN mapping. The complete list of criteria is presented in Table 1. The studies defined 3 populations of patients: a population of all enrolled patients, an intent-to-treat (ITT) population, and the safety population. The ITT population consisted of all enrolled patients receiving both [^99m^Tc]tilmanocept and VBD and who had at least one histologically confirmed lymph node stained by VBD. This was the population in which the concordance of [^99m^Tc]tilmanocept to VBD could be assessed, hence the ITT designation. Analyses of pathology rates were based on findings from any patient injected with [^99m^Tc]tilmanocept and from whom lymph nodes were removed. Drug safety was based on the safety population, consisting of all patients who received [^99m^Tc]tilmanocept whether or not they received VBD.

**Table 1:** Study enrollment criteria

Inclusion criteria
Confirmed presence of primary breast cancer
Candidate for surgical intervention, with lymph node mapping being part of the surgical plan
At least 18 years of age at time of consent
ECOG performance status of grade 0–2
If female, either negative pregnancy test within 72 h prior to [^99m^Tc]tilmanocept administration, having been surgically sterilized, or postmenopausal for at least 1 year prior to start of study
Pure ductal carcinoma in situ (DCIS) or noninvasive carcinoma with lymph node biopsy as part of the surgical plan

Exclusion criteria
Pregnancy or lactation
Clinical or radiological evidence of metastatic cancer, including palpably abnormal or enlarged lymph nodes
Known hypersensitivity to Lymphazurin or patent blue V
Participation in another investigational drug study
Bilateral primary breast cancers or multiple breast tumors
Prior surgical breast surgery (e.g., axillary surgery, implants)
Scheduling for bilateral mastectomy if bilateral SLN mapping is required
Preoperative radiation therapy to the affected breast or axilla

Site qualification required that all participating surgeons and investigators had performed at least 30 SLN procedures with a radiopharmaceutical within the past 90 days prior to initiation of the trial.

### Procedures

Patients received 3.0 nmol of [^99m^Tc]tilmanocept (50 μg). Patients scheduled for surgery on the same day as the injection received ~0.5 mCi of [^99m^Tc]tilmanocept. Patients scheduled for “next-day” surgery received ~1.0–2.0 mCi of [^99m^Tc]tilmanocept. The radiopharmaceutical was administered using one of the following routes of administration: intradermal, subareolar, or peritumoral. For a given patient, isosulfan blue was injected after [^99m^Tc]tilmanocept at the time of surgery using the same injection route as the radiopharmaceutical. Injections of [^99m^Tc]tilmanocept were not accompanied by either topical application of or coinjection with local anesthetics.

Intraoperative identification of SLNs was based on three criteria. The first criterion was visual identification of VBD in a node and/or the afferent lymphatic channel; all blue-stained nodes were removed and designated as “blue.” Observation of radioactivity in a lymph node, indicating the localization of [^99m^Tc]tilmanocept, formed the basis of the second criterion. Background radioactivity levels were measured using a handheld intraoperative gamma detector/probe (programmed to read out in counts per second) at the skin surface well away (not less than 20 cm) from the injection site. The background count rate was recorded directly from the display of the gamma detection system. The standard deviation of the background was calculated as the square root of the total number of counts acquired during the measurement, which equaled the background count rate, in counts per second, times the duration of the counting time, in seconds. To qualify as a hot node, the intraoperative counts in the node had to exceed the background count (using either one 10-second count or the average of three 2-second counts, with background measured directly on the patient ≥20 cm from the primary site) plus 3 times the standard deviation of the background and exceed 25 counts per second. The “3-sigma rule,” was selected for this study based on statistical rigor; using this rule provides 99.7 % certainty that detection of the “hot” node did not occur due to chance. Blue nodes were evaluated for radioactivity prior to and after excision. The third SLN identification criterion was based on appearance and feel; visibly or palpably abnormal lymph nodes were designated as palpable masses and were excised regardless of agent localized. Mapping was considered complete when all nodes meeting any of the 3 criteria had been removed and all remaining nodes in the basin were designated as negative based on these criteria. All excised nodes and tissues underwent histopathological evaluation that included hematoxylin and eosin staining and immunohistochemical analysis. Based on tissue analysis, tissue organization was confirmed (lymph node) and pathology status was determined.

### Safety Data

The safety endpoint was monitored by performing follow-up safety labs, EKGs, and physical examinations 6–30 h postinjection and comparing results with baseline values. All patients were monitored for adverse events. Adverse events were considered severe if they resulted in death, represented a life-threatening reaction, or required prolonged or readmission to the hospital. The principal investigator for each site was responsible for determining if adverse events were definitely not, probably not, possibly, probably, or definitely related to [^99m^Tc]tilmanocept administration. The safety data was analyzed with parametric statistics if the test results were reported as continuous variables.

### Statistical Plan

The primary efficacy endpoint in both studies was the concordance of radioactive lymph nodes identified with [^99m^Tc]tilmanocept and those identified with VBD. Concordance of [^99m^Tc]tilmanocept with VBD was defined as the number of blue-stained nodes that were detected by [^99m^Tc]tilmanocept, divided by the number of blue-stained lymph nodes. A supportive secondary endpoint was the patient concordance rate defined as the number of patients for whom all nodes detected by VBD were also detected by [^99m^Tc]tilmanocept, divided by the number of patients with at least one blue-stained lymph node (ITT population). Other secondary efficacy endpoints included the proportions of all removed lymph nodes that were detected by [^99m^Tc]tilmanocept and/or VBD. The proportions of pathology-positive lymph nodes that were blue and/or radioactive were evaluated, including calculation of failed detection rates for the imaging agent. Concordance (*R*) was tested in a pooled analysis with the hypothesis H0: *R* ≤ 0.90 versus Ha: *R* > 0.90. A 95 % confidence interval for the concordance rate was calculated using a large sample normal approximation.

### Role of the Funding Source

Navidea Biopharmaceuticals (Dublin, OH) sponsored these trials and supplied tilmanocept kits for radiolabeling to each study site. STATKING Clinical Services (Fairfield, OH), an independent data analysis group, facilitated independent data auditing and analyses.

## RESULTS

### Patient Population

A total of 152 women with breast cancer were enrolled in the 2 trials, and 149 were injected with [^99m^Tc]tilmanocept (3 patients withdrew prior to injection). Therefore, 149 made up the safety population (all patients receiving [^99m^Tc]tilmanocept). All breast cancer patients were female. The safety population’s average age was 58.0 years (range, 31–84 years). Specific demographics of the various subpopulations of the safety population are listed in Table 2. One patient injected with [^99m^Tc]tilmanocept was not injected with VBD. All surgeons involved in both trials had at least 10 years of experience performing sentinel lymph node mapping of breast cancer.

**Table 2:** Demographics of patients receiving [^99m^Tc]tilmanocept

Characteristic	*N* = 149
Male, female	0, 149
Age, median (range) (years)	58.0 (31–84)
Weight, median (range) (lb)	154.8 (70–325)
Race	
White	132
Black	6
Asian	10
Native Hawaiian or other	1
Pacific Islander	0
Ethnicity	
Non-Hispanic	131
Hispanic	10
Unknown	8
Tumor stage	
TX	1
Tis	14
T1	104
T2	27
T3	1
T4	2

Includes 2 patients who never received vital blue dye and are excluded from concordance analyses

*N* number of patients injected with tilmanocept

### Intraoperative Node Identification

There were 148 patients injected with both [^99m^Tc]tilmanocept and VBD and who underwent surgery, of which 131 (88.5 %) had at least one blue node (ITT population) and 146 (98.6 %) had at least one radioactive node (Table 3). The observation that [^99m^Tc]tilmanocept detected SLNs in more patients than VBD (98.6 vs 88.5 %) was statistically significant (*p* < 0.0001). A single breast cancer patient had two palpable lymph nodes missed upon pre-enrollment review (later found to contain cancer) that were neither blue-stained nor radioactive. Prospectively, per protocol, these nodes were defined as SLNs.

**Table 3:** Intraoperative mapping results

Measurement	Pooled analysis
Patients injected with both vital blue dye and [^99m^Tc]tilmanocept	*N* = 148
Detection rate,^a^ No. of patients (%)	
Blue (ITT patients)	131 (88.5)
Radioactive	146 (98.6)
*p* value, blue vs. radioactive^b^	<0001
Detection rate, No. of nodes (%)	326
Blue	209 (64.1)
Radioactive	320 (98.2)
*p* value, blue vs radioactive^b^	<0001
Average blue nodes per ITT patient, No.	1.60
ITT patients with ≥1 radioactive node, No. (%)	131 (100.0)
Average radioactive nodes per patient, No.	2.19
Patients with ≥1 blue node, No. (%)	131 (89.7)
Pathologically positive lymph nodes	33
Blue and radioactive, No. (%)	25 (75.8)
Blue and not radioactive, No. (%)	0 (0.0)
Radioactive and not blue, No. (%)	6 (18.2)
Not blue and not radioactive, No. (%)^c^	2 (6.1)
Pathologically positive node detection rate	
Vital blue dye, No. (%)	25 (75.8)
[^99m^Tc]tilmanocept, No. (%)	31 (93.9)
*p* value^b^	0.0312
Failed detection rate by node	
Vital blue dye (%)	24.24
[^99m^Tc]tilmanocept (%)	6.06
*p* value^b^	0.0312
Patients with missed positive nodes^d^	
No. of patients with at least 1 pathologically positive node	27
≥1 node missed by vital blue dye, No. (%)	6 (22.2)
≥1 node missed by [^99m^Tc]tilmanocept, No. (%)^c^	1 (3.7)

*N* number of patients injected with both [^99m^Tc]tilmanocept and vital blue dye and who underwent surgery, *blue* blue-stained lymph node, *radioactive* radioactive lymph node (see Methods section)

^a^At least 1 lymph node

^b^2-sided *p* value for exact McNemar’s test

^c^Both nodes from the same patient, both nodes were palpably enlarged and tumor replaced

^d^From patients injected with both [^99m^Tc]tilmanocept and vital blue dye and who underwent surgery

There were 326 lymph nodes examined in these studies. Of 131 patients with at least 1 blue node (ITT population), an average of 1.60 blue nodes were detected per ITT patient for a total of 209 blue nodes. Of 146 patients with at least one radioactive node, an average of 2.16 radioactive nodes were detected per patient for a total of 320 radioactive nodes. Also, 15 patients with at least one radioactive node had no blue nodes.

**Fig. 2:**
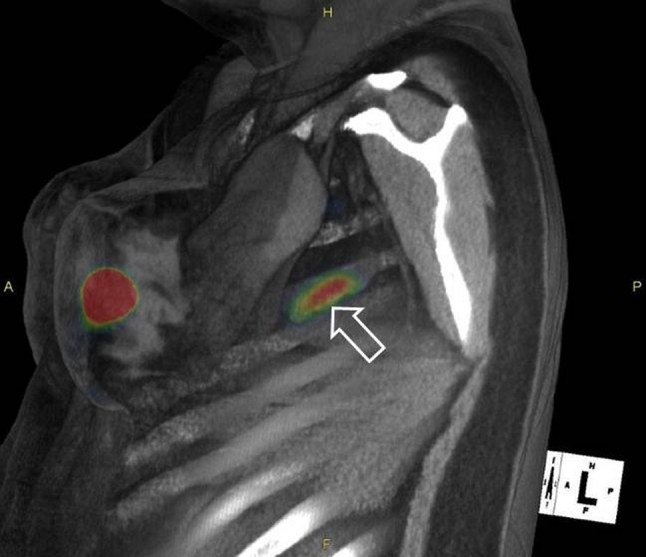
Lymphoscintigraphy of a 35-year-old woman with carcinoma in situ of the left breast showing 2 intense foci of noted [^99m^Tc]tilmanocept localization within the left axilla. An intradermal injection (0.4 mL, 0.5 mCi, 3.0 nmol) of [^99m^Tc]tilmanocept was administered to the upper left quadrant of the left breast. The SPECT/CT image is a fused sagittal cross section acquired 1 h postinjection, which visualizes a sentinel lymph node (*arrow*) and the injection site. At 5 h after injection, 3 blue and hot lymph nodes (6,724 cps, 1,477 cps, 167 cps) were detected at surgery and excised. Pathologic examination revealed 1 histologically positive lymph node (blue with 6,700 cps, 1.7 × 1.3 × 0.7 cm) and 2 negative lymph nodes

Figure 2 is a fused sagittal cross section acquired by SPECT/CT imaging at 1 h postinjection. The cross section visualizes a sentinel lymph node (arrow) and the injection site. At 5 h after injection, 3 blue and hot lymph nodes (6,724 cps at arrow, 1,477 cps, 167 cps) were detected at surgery and excised. Pathologic examination revealed 1 pathology positive lymph node (blue with 6,724 cps, 1.7 × 1.3 × 0.7 cm) and 2 pathology negative lymph nodes.

### Concordance of [^99m^Tc]Tilmanocept and Vital Blue Dye

The primary efficacy endpoint in both studies was the concordance of radioactive lymph nodes identified with [^99m^Tc]tilmanocept and those identified with VBD. Of 209 blue lymph nodes identified in the ITT population, 207 (99.04 %, 95 % CI = 96.59–99.88 %) were also radioactive (Table 4). This concordance rate was statistically significant (*p* < 0.0001), convincingly rejecting the null hypothesis of ≤90 %. For 129 of 131 patients in the ITT population, all blue nodes were also radioactive for a pooled patient concordance rate of 98.47 %.

**Table 4:** [^99m^Tc]Tilmanocept concordance with vital blue dye

	Concordance rate			
	Patient^a^		Lymph node^b^	
	Total *N*	Concordant *N* (%)	Total SLN	Concordant SLN (%)
Pooled analysis	131^c^	129 (98.47)	209	207 (99.04)
95 % CI^d^		(94.59–99.81)		(96.59–99.88)
*p* value^e^		0.0002		<0.0001

*N* number of ITT patients, *SLN* number of lymph nodes

^a^Patient concordance rate: the number of patients for whom all nodes detected by vital blue dye were also detected by [^99m^Tc]tilmanocept divided by the number of patients with at least 1 blue-stained lymph node

^b^Lymph node concordance rate: the number of blue-stained nodes that were detected by [99mTc]tilmanocept divided by the number of blue-stained lymph nodes

^c^The intent-to-treat population

^d^95 % exact binomial confidence interval for pooled analysis proportion

^e^2-sided *p* value for exact 1-sample binomial test of pooled analysis concordance against null hypothesis of ≤90 %

### Pathology Findings

Of the 148 injected with both [^99m^Tc]tilmanocept and VBD and who underwent surgery, a subset analysis was performed on the 27 patients in which 1 or more pathology-positive lymph nodes were detected (Table 3). A total of 33 pathology-positive nodes were detected in these 27 patients. All metastases were identified by hematoxylin and eosin staining and were greater than 2 mm. Except for 2 cancer-containing palpable lymph nodes found in 1 patient in the first study (mentioned previously), all pathology-positive lymph nodes were radioactive. Conversely, while [^99m^Tc]tilmanocept detected 31 of 33 cancer-containing lymph nodes (93.9 %), VBD identified only 25 of 33 such nodes (75.8 %), missing the involved pathology-positive lymph node(s) in 6 patients (22.2 %). In fact, 2 patients (2 of 148; 1.35 %) had their nodal disease status upstaged by [^99m^Tc]tilmanocept findings alone; no patients were upstaged by VBD alone. Thus, [^99m^Tc]tilmanocept exhibited a higher detection rate for disease-positive lymph nodes than VBD on a per node and per patient basis. The nodal failed detection rate over both studies for [^99m^Tc]tilmanocept was 6.06 %, whereas VBD’s nodal failed detection rate was a significantly greater at 24.24 % (*p* = 0.0312).

### Adverse Events

No patient deaths were reported on study. A total of 74 adverse events occurred in 49 patients in the safety population of breast cancer patients receiving [^99m^Tc]tilmanocept (*N* = 149). The most common events included nausea, seroma, and urinary tract infection. There were 3 serious adverse events reported from 3 patients (modified radical mastectomy, herpes zoster ophthalimic, and cellulitis), none of which was deemed “probably” or “definitely” related to [^99m^Tc]tilmanocept. The remaining adverse events were generally considered mild. There were no immediate or delayed hypersensitivity reactions to [^99m^Tc]tilmanocept. None of the events that were considered clinically significant was related to the administration of [^99m^Tc]tilmanocept. Even though local analgesics were not used in conjunction with administration of the [^99m^Tc]tilmanocept, only 2 patients reported injection site pain or slight breast pain during injection related to study drug. Both of these adverse events were considered mild and resolved the same day as onset.

## DISCUSSION

In this pooled analysis of breast cancer patients participating in 2 highly similar phase 3 trials, [^99m^Tc]tilmanocept’s identification of lymph nodes was highly concordant with identification by VBD. Of the 209 lymph nodes identified by VBD, 207 were also identified by [^99m^Tc]tilmanocept (*p* < 0.0001). Interestingly, VBD failed to identify a SLN in 17 of 148 breast cancer patients (11.5 %). This was less than the reported failure rate for VBD.^17^ Within these 17 patients, [^99m^Tc]tilmanocept identified at least 1 node in all but 2; notably, these patients also had no blue nodes. There were significantly (*p* < 0.0001) more radioactive nodes (SLN = 320) than blue nodes (SLN = 209). Of 320 radioactive nodes, only 207 (64.69 %) were also blue. Similarly, in only 78 of 146 patients (53.42 %) with at least 1 radioactive node were all the radioactive nodes also blue. Our findings are consistent with those of Krag and coworkers, where the isotope was better than VBD in all aspects.^18^

[^99m^Tc]Tilmanocept’s ability to identify more cancer-containing nodes than VBD (31 vs. 25, *p* < 0.0312) demonstrates that [^99m^Tc]tilmanocept provides a benefit in intraoperative lymphatic mapping beyond that provided by VBD alone. Recent reports that local recurrence and survival are not affected by full node dissection after positive sentinel node biopsy in breast conservation patients accentuates the importance of the performance of the [^99m^Tc]tilmanocept SLN mapping agent in these studies and points to the need to accurately assess lymph nodes in order to optimize postsurgery management of the patients.^1^^,^ 19 ^,^ 20 Our findings suggest, then, there is significant clinical utility in knowing which patients have multiple positive sentinel nodes.

Entrance of a patient into the clinical trial was at the discretion of the surgeon. As long as the patient did not have metastatic disease, clinically positive lymph nodes, or any of the exclusion criteria listed in Table 1, the patient was eligible for the study. If the 2 T4 patients were excluded the concordance would be slightly higher and the performance of VBD would appear to be better. This is because in one of the patients VBD failed to stain a disease-positive lymph node. Two patients were stage T4. Both patients had 2 “hot” lymph nodes. Both nodes of one patient were disease-positive with 1 of the lymph nodes not stained blue. The other patient had a “hot” and “blue-stained” lymph node that was disease-positive; the second “hot” lymph node was disease-negative and not stained blue. Based on criteria other than T staging, for example, N0, M0, there is no reason to exclude such patients.

There is a growing discussion as to whether or not a complete node dissection is necessary.^3^^,^ 19 ^,^ 21 [^99m^Tc]Tilmanocept identified numbers of patients who had additional pathology-positive sentinel nodes, while VBD did not. A study site, University of California, San Diego, had 20 breast cancer patients with pathology-positive nodes identified with at least 1 radioactive and/or blue sentinel node; 16 had only a single radioactive and blue positive sentinel node. Also, four patients had additional radioactive pathology-positive nodes that were not blue, deeming them “multinode positive patients.” The indication that [^99m^Tc]tilmanocept may be able to better identify multinode pathology-positive patients may be important to the future of breast cancer treatment. Specifically, extended field radiation therapy will be offered to these patients if not full node dissection. To the extent that [^99m^Tc]tilmanocept accumulates at that the target tissue based on a specific biochemical interaction (CD206 receptor-targeted), this molecule represents the current generation of imaging technology and is specifically designed to match the biochemical requirements of the clinical problem. Thus, the increase in found disease represents a notable finding related to the SLN procedure, especially where this is translated to a large patient population. If blue dye alone were used, the addition of tilmanocept could potentially change the diagnosed number of positive nodes in 1.35 % of our nation’s 190,000 or so breast cancer patients, which is roughly 2,000 patients per year. Since adjuvant decisions are now often based on sentinel node biopsy results alone, accurately knowing those patients who have multiple positive nodes may change radiation and systemic therapy choices.

Vital blue dye was chosen as the comparator in these studies because it is routinely used in current practice to identify SLNs during breast cancer surgery and has a history as a validation agent for SLN biopsy.^6^^,^ 22 Radioactive colloids have been used off-label with the intent to augment VBD.^7^^,^ 8 ^,^ 23 However, [^99m^Tc] sulfur colloid injection (SCI) has recently been granted FDA approval in the United States for lymphatic mapping during breast cancer operations only. Nevertheless, radiocolloids remain an unstandardized SLN mapping agent, mitigating their utility as a valid comparator agent. VBD usage permitted a rigorous, within-patient experimental design, significantly increasing the statistical power. There is a possibility despite finding one blue node and finding several hot nodes, that a rare additional aberrant blue node was missed somewhere. It is also possible when no blue node was identified at all and several hot nodes were found, that again a distant blue node may have been overlooked. However, most of the positive axilla did go on to full node dissection, and no further blue nodes were identified.

These phase 3 studies provided a basis with which to compare the performance of the radiolabeled colloids via meta-analysis. A recent review of SCI was based on a meta-analysis of 15 studies involving 9,213 breast cancer patients receiving both SCI and VBD.^24^ In the meta-analysis, SCI and VBD detected at least 1 lymph node in 94.1 and 85.1 % of patients, respectively. In the current studies, [^99m^Tc]tilmanocept and VBD detected at least 1 lymph node in 98.6 % (146 of 148) and 88.5 % (131 of 148) of patients, respectively. Lastly, we constructed a meta-analysis of these phase 3 trials and contrasted the results against the recently published selected performance data of Nanocoll.^25^ Tilmanocept had superior SLN localization rate (99.9 vs. 95.1 %) and a higher degree of localization (2.16 vs. 1.66 SLN per study) (*p* < 0.0001). Such comparisons will be duplicated with larger sample sizes if [^99m^Tc]tilmanocept becomes commercially available.

These phase 3 trial and the previous phase 1 and 3 studies provided preliminary data regarding the performance of [^99m^Tc]tilmanocept compared with the current SLN agents. First, [^99m^Tc]tilmanocept is a wholly standardized synthetic molecule with a defined molecular structure that permits rapid uptake into lymph nodes. When imaged within 10 min postinjection, at least 1 “hot” lymph node was detected in 98 % of the early images. Second, based on physician use reports, the administration of [^99m^Tc]tilmanocept is associated with significantly less pain and discomfort than for radiolabeled colloids. Third, based on the contrasts of dosimetry and preoperative imaging, [^99m^Tc]tilmanocept exits its injection site significantly quicker than radiolabeled colloids, creating less shine-through, thus facilitating better step-down for observation of SLNs that reside near injection sites.^15^ Finally, both in vivo imaging and in vitro analysis of [^99m^Tc]tilmanocept’s receptor binding properties indicate it binds specifically to SLNs and does not move downstream to distal lymph nodes, permitting high confidence that a “hot” lymph node found during a “next-day” surgery is a sentinel lymph node.^9^^,^ 15 ^,^ 26 This factor also allows the patient to be imaged as early as 10 min and as late as 24 h or more after injection. These attributes will translate into greater scheduling flexibility in nuclear medicine and operating room without sacrificing either short-term or long-term SLN detection success.

[^99m^Tc]Tilmanocept is a modern radiopharmaceutical designed on a chemical platform that permits future development.^26^ Technetium-99m-labeled antimony sulfide colloid and sulfur colloid, both of which were developed more than 35 years ago, cannot be chemically modified.^27^^,^ 28 The chemical design of tilmanocept permits the facile attachment of additional imaging reporters, such as fluorescent dyes.^29^^,^ 30 Routine optical imaging via robotic-assistance or intraoperative hand-held imaging will require the covalent attachment of the fluorescent tag to the molecular imaging agent.^31^^,^ 32 Optical imaging would facilitate intraoperative administration, which may be more compatible with SLN mapping of lung, GI, and GU cancers. The DTPA chelators of tilmanocept (Fig. 1) permit radiolabeling with different radioisotopes, such as gallium-68 or indium-111 or paramagnetic atoms.^33^ Gallium-68, which is a generator-produced positron-emitter, enables PET/CT hybrid imaging.^34^ PET imaging provides higher sensitivity and spatial resolution, as well as greater scatter and attenuation correction than SPECT. Hybrid imaging with PET/CT may prove to be more compatible for preoperative pelvic imaging of prostate cancer. Lastly, Nanocoll is a microcolloid of human serum albumin and must be tested for antibodies to HIV and hepatitis C, as well as hepatitis B surface antigen.^35^ Rigorous testing and increased regulatory compliance add expense to the manufacturing of Nanocoll and substantially decreases the likelihood that modifications for performance improvements will be commercialized. Tilmanocept is not derived from blood and therefore can be synthesized at very high scales without continual monitoring for new biological “threats” from the human blood supply.

There were two reasons for using the “3-sigma” rule. First, the “3-sigma” rule is based on a statistical rationale and is used extensively in the radiation sciences to define a criteria known as the “Minimum Detectable Activity.”^36^ When a measurement is greater than background by 3 times the standard deviation of the background, the probability that the measurement is truly different than background is greater than 99.7 %. The measurement of radioactivity is a counting process that follows a Poisson distribution, which occurs when the process is the result of many attempts and very few successes. The standard deviation of a Poisson distribution is the square root of the measurement. Consequently, the Minimum Detectable Activity for a sentinel lymph node is 3 times the square root of the background count rate, which can be obtained by a single measurement. Second, the “10 %” was empirically established for radiocolloids and may not be applicable to a nonparticulate receptor-targeted radiopharmaceutical.^18^ Due to its small size (7 nm) and high receptor avidity, tilmanocept delivers significantly more radioactivity to the sentinel lymph node and clears from the injection site 5-fold faster than sulfur colloid. For example, the UCSD breast cancer patients were studied with a protocol that used a single intradermal injection of 0.1 ml. A total of 95 patients had at least 1 “hot” lymph node. The count rate of the “hottest” lymph node from each patient ranged from 375 cps to an excess of 999,999 cps, which is the readout maximum of the Neo200 counting system. Seven lymph nodes exceeded this count rate. The median count rate of the “hottest” lymph node was 15,738 cps. Appling the “10 % rule” when the “hottest” node has 15,000 cps would permit the surgeon to stop if the surgical bed had a count rate of 1,500 cps or less. This would risk leaving behind a lymph node that could be 1,500 cps and by radiocolloid standards would be considered very “hot.” Such a lymph node would be almost 3 orders-of-magnitude higher than background and would more than satisfy the “3-sigma rule,” which was our statistical basis for defining a radioactive signal and, hence, a sentinel lymph node.

A sentinel lymph node definition based on the technical criteria of “Minimal Detectable Activity” may not provide the most efficient “stopping rule” for the mapping procedure. Although a “stopping rule” for a receptor-targeted agent such as [^99m^Tc]tilmanocept will require a separate study and may not be at all similar to the rule used for particulate radiotracers, a comparison of our results to the “10 % rule,” which is used for unfiltered and filtered [^99m^Tc]labeled sulfur colloid may be instructive.^18^^,^ 37 ^–^ 40 The “10 % rule” and the “3-sigma rule” identified the same number of patients (*n* = 146) with at least 1 “hot” lymph node. Consequently, the per-patient concordance rate was the same using both rules. Additionally, defining the sentinel node by the “10 % rule” did not alter the pathology status on a per-patient basis. Also, 17 fewer sentinel lymph nodes were defined as “hot” based on the “10 % rule.” Of these, 11 nodes were blue, and 2 of the 11 were pathology-positive.

In summary, these studies demonstrated that SLN detection by [^99m^Tc]tilmanocept is highly concordant to SLN detection by VBD. [^99m^Tc]Tilmanocept detected more SLNs than VBD, and these additional SLNs enhanced the detection of clinically important nodal metastases. Given these results, this agent is safe and provides a modern radiopharmaceutical for future advances in sentinel lymph node mapping.
